# KRAS Inhibition in Pancreatic Ductal Adenocarcinoma

**DOI:** 10.3390/jcm15020873

**Published:** 2026-01-21

**Authors:** Roshini Pradeep, Nooredeen Jamal Isbeih, Freya F. Abraham, Ehsan Noori, Zachary P. Yeung, Madappa N. Kundranda

**Affiliations:** 1 Department of Hematology and Oncology, University of Arizona College of Medicine—Phoenix, Banner MD Anderson Cancer Center, Gilbert, AZ 85234, USA; 2 University of Arizona College of Medicine—Phoenix, Phoenix, AZ 85004, USAehsan.noori@bannerhealth.com (E.N.)

**Keywords:** KRAS, pancreatic ductal adenocarcinoma, RAS mutation, KRAS G12C inhibitor, switch region, crosstalk

## Abstract

KRAS alterations are a hallmark of pancreatic ductal adenocarcinoma (PDAC) found in >90% of tumors. This review examines the historical evolution of the understanding of RAS and its central role in PDAC biology. We summarize the various downstream effectors, feedback loops, and resistance mechanisms that play a pivotal role in PDAC oncogenesis. Our review explores the early development of covalent inhibitors of KRAS G12C and efforts at specific inhibition of other codons and newer approaches of targeted protein degradation. We subsequently summarize the development of panRAS inhibitors and allosteric and switch-region targeting before focusing on rational therapeutic blockade of crosstalk and upstream signaling, with attention to synthetic lethality approaches transitioning from preclinical to early-phase in-human clinical trials. This review elaborates on ongoing KRAS-specific siRNA research and evolving KRAS-directed immunotherapies. We conclude by outlining the current KRAS clinical trial landscape and future areas of investigation.

## 1. Introduction

Gastrointestinal malignancies account for 26% of worldwide cancer incidence and over 34% of all cancer-related mortality. Pancreatic cancer represents 2.6% of all new cancer cases worldwide and was responsible for 467,005 deaths globally in 2022 [1]. Among pancreatic cancers, Pancreatic ductal adenocarcinoma (PDAC) represents up to 85% of such cases [2]. Curative intent surgery is possible only in 20% of cases [3]. Systemic chemotherapy options are limited with only five active drug classes, including 5-fluorouracil (5-FU), oxaliplatin, irinotecan, gemcitabine, and nab-paclitaxel, which confer a dismal overall median survival in metastatic patients of under a year. There is an unmet need for additional therapeutics in this patient population, prompting growing interest in targeted molecular therapies [4,5,6].

**Figure 1:**
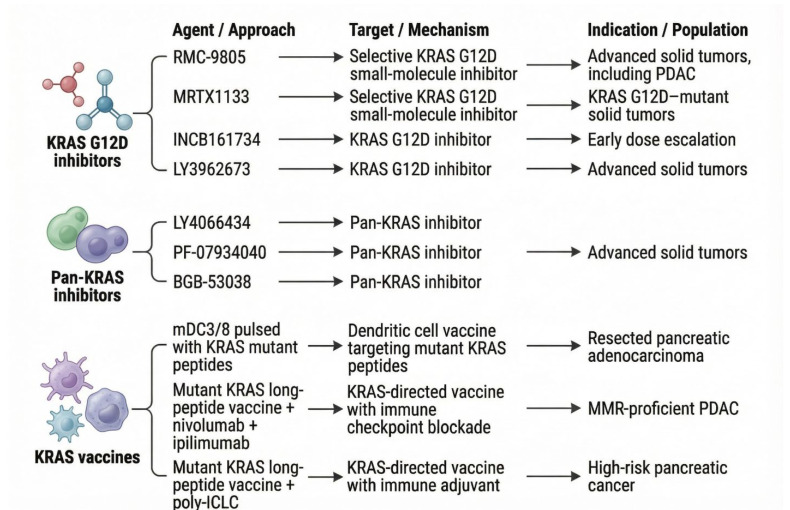
Graphical depiction of the prevalence of RAS mutations found in specific cancers. Description: RAS mutations were found to be particularly prevalent in pancreatic, colon, and lung adenocarcinomas, with 88.4%, 54.7%, and 33.2% of cases harboring mutated RAS, respectively. Special emphasis was placed on KRAS mutations in pancreatic cancer, where KRAS mutation was identified in 88% of cases.

RAT Sarcoma Virus proto-oncogenes were first recognized in the early 1980s. Following the discovery of Harvey Sarcoma Virus (Ha-SV) and Kirsten Sarcoma Virus (Ki-SV), retroviruses that induce tumors in mice, HRAS and KRAS proto-oncogenes in human cancers were later described. KRAS was the first human oncogene characterized. Those discoveries launched the field of molecular oncology [7], and soon after, NRAS was identified in neuroblastoma cells [8]. Hence, the RAS gene family comprises KRAS 4A, KRAS 4B, HRAS, and NRAS [9]. RAS mutations are predominant in pancreatic, colorectal, and lung adenocarcinomas, while NRAS is more common in melanomas and thyroid malignancies, HRAS is more prevalent in head and neck squamous cell carcinomas and urothelial carcinomas of the bladder [10]. In a review of the largest cancer genomics databases available (Catalogue of Somatic Mutations in Cancer (COSMIC), The International Cancer Genome Consortium (ICGC), The Memorial Sloan Kettering Cancer Centre (cBioPortal), and The Cancer Genome Atlas (TCGA), RAS mutations were found to be particularly prevalent in pancreatic, colon and lung adenocarcinomas with 88.4%, 54.7% and 33.2% of cases harboring mutated RAS, respectively [Figure 1]. In PDAC, KRAS mutations were identified in 88.0% of cases, contributing to 48,000 new cases in the US annually. NRAS was mutated in 0.4% of cases. Overall, KRAS accounts for approximately 2,475,000 cases globally per year, and despite the lower percentages of NRAS/HRAS involvement, they remain significant, accounting for nearly 900,000 cancer cases annually. Notably, KRAS mutations are common in PDAC and are founder oncogenic mutations that drive PDAC biology.

Rare codons account for the overall predominance of KRAS-mutated cancers [11,12]. In pancreatic adenocarcinoma, 84% and 62% of cases harbored KRAS and TP53 mutations, respectively [13]. Mutation hotspots are distinct among RAS isoforms. Five mutational hotspots (G12D, G12V, G12C, G13D, and Q61) account for the majority of all RAS-mutated patients, all occurring in the effector lobe of the protein. The G12X codon mutation accounts for over 80% of KRAS mutations in cancer cells. KRAS G12D is the most common mutation in pancreatic adenocarcinoma [10]. Most PDACs originate from Pancreatic Intraepithelial Neoplasms (PanINs) [14]. Molecular profiling studies revealed that more than 95% of PanINs harbored KRAS mutations, with the majority being in codon 12 [15], supporting KRAS mutations as the driving event in developing PDAC [16]. The most frequent mutation was observed in G12D (31–60%), G12V (23–26%), G12R (10–21%), and less frequent mutations in Q61H (4–7%) and G12C (1–2%) [17]. Building on evidence that KRAS is foundational to the development of the hostile tumor microenvironment (TME) and a driver of therapeutic resistance, efforts have been made to target KRAS to improve outcomes [10]. We will explore the targeting landscape, review the literature, and provide a scoping review of multiple mechanisms of targeting KRAS and other related signaling effectors.

### 1.1. Scope and Organization of the Review

There is a large body of data on KRAS mutations and the targeted treatments available for them. There is well-described data on the role of KRAS inhibitors and the resistance pathways in various cancer types. The scope of this review is to focus specifically on pancreatic ductal adenocarcinoma. We have discussed the biology of KRAS, signaling pathways, KRAS inhibitors, and combination therapies, drawing on precise data that have shaped recent advances in pancreatic adenocarcinoma. The clinical trials included were carefully selected from ClinicalTrials.gov based on studies that had recently completed or were actively recruiting. Most studies are Phase 1 or Phase 2, with only a few Phase 3 trials, which are discussed in this review. In this review, we aim to summarize the data most clinically relevant to PDAC KRAS biology.

### 1.2. Fundamentals of RAS Biology in Cancer Cells

RAS gene mutations were previously estimated to occur in almost a third of all cancers. KRAS mutations are common in adenocarcinomas and account for 14% of all adenocarcinomas, regardless of cancer site [13]. Large genetic databases revealed strong coupling between specific RAS gene isoform mutations and certain cancers. KRAS mutations commonly co-occur with other mutations. RAS proteins cycle between an inactive GDP-bound state, referred to as “RAS-Off,” and an active GTP-bound state, referred to as “RAS-On” [18]. RAS mutations impair GTP-GDP cycling through various mechanisms, thereby increasing signaling through downstream effectors [19]. A membrane-bound GTPase relays the signal to the downstream effectors via the RAF/MAPK, PI3K, and RAL-GDS pathways. Those pathways regulate cellular proliferation, survival, differentiation, and growth [9]. Activating mutations in the RAS proto-oncogenes are common in cancer, occurring in 15–19% of all human tumors, depending on the genomic database used [20].

Several studies have shown that KRAS-driven PDAC growth is heavily mediated by the RAF/MEK/ERK MAPK signaling cascade. Activated ERK can then create a complex phosphoproteome comprising thousands of cytoplasmic and nuclear proteins. These can further alter cell signaling and promote PDAC growth. PI3K acts as another major effector of KRAS, converting phosphatidylinositol-4,5-bisphosphate (PIP2) to phosphatidylinositol-3,4,5-triphosphate (PIP3) and then activating AKT1-3 serine/threonine protein kinases. Activation of these kinases can then signal mTOR to promote further cell proliferation [1,21]. While not their primary function, these signaling pathways contribute significantly to tumor desmoplasia, creating a tumor microenvironment (TME) that is poor in vasculature, oxygenation, and a barrier to drug penetration and efficacy [22].

KRAS4B has been more extensively studied [13]. The four RAS protein isoforms are highly homologous with a shared 82–90% amino-acid sequence identity; they share identical amino acid (aa) sequences in the N-terminal “effector lobe” that comprises the G domain that is responsible for GTP binding and hydrolysis. In contrast, they share a few similarities in the C-terminal (hypervariable region). There is growing interest in divergence between the isoforms in the allosteric lobe, as it has a distinct role in subcellular localization and effector utilization [8,15,23,24].

### 1.3. Downstream Effectors, Feedback Loops, Resistance Mechanisms

**Figure 2:**
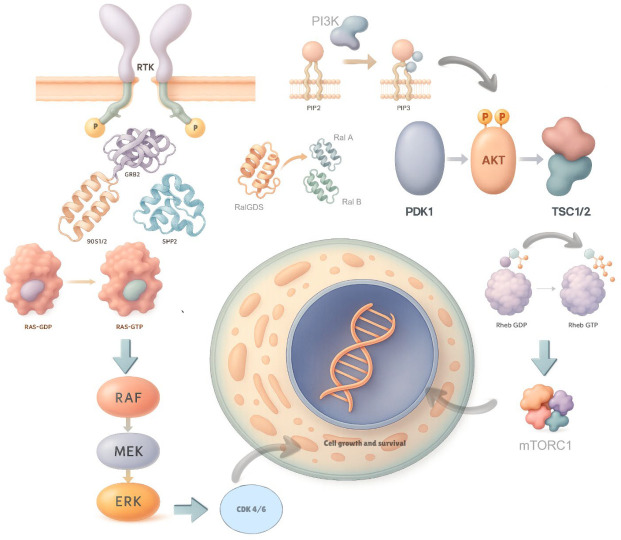
Upstream and downstream RAS signaling networks in pancreatic ductal adenocarcinoma (PDAC). Description: This schematic illustrates KRAS-driven signaling pathways downstream of receptor tyrosine kinases (RTKs), highlighting activation of the RAF–MEK–ERK (MAPK), PI3K–AKT–mTOR, and RalGDS–RalA/B axes. Together, these interconnected pathways regulate cell growth, survival, and proliferation, with key nodes such as AKT, mTORC1, and CDK4/6 shown as critical effectors relevant to oncogenic signaling in cancer.

The main downstream effector pathways of KRAS are RAF/MEK/ERK, PI3K/PTEN/AKT, and RAL-GDS pathways. The active form of RAS binds three RAF kinases (A-RAF, B-RAF, and C-RAF) that further cause MEK1/2 phosphorylation, leading to MAPK activation. This facilitates activation of ERK1/2 mitogen-activated protein (MAP) kinases, which have numerous downstream effects in transcription, cell cycle, survival, and differentiation [16]. B-RAF is considered the dominant MEK activator, as evidenced by its constitutive kinase activity and its oncogenic role in human cancers [23,25,26,27,28]. PI3K is the other major pathway. PI3Ks substrate 3-phosphoinositide-dependent kinase (PDK1) activates AKT, which in turn regulates downstream processes that control the cell cycle, metabolism, apoptosis, and DNA repair. Also, Rapamycin-insensitive mammalian target of rapamycin (mTOR) complex (mTORC2) plays a crucial role in AKT activation [29]. PI3K’s oncogenic role has been extensively studied. For example, PDK1 deletion was found to block the development of KRAS G12D-induced PDAC in vivo [30,31], and PI3K mutations were observed in WT KRAS but are mostly concurrent with mutant KRAS-induced PDAC [32,33,34] [Figure 2].

RAS effector pathways are interlinked beyond their independent signaling, with complex cross-inhibitory and activating mechanisms [32,33]. Moreover, negative feedback loops exist within each pathway; for example, ERK activation results in RAF/MEK1 inhibition [34], and similarly, substrates of the PI3K pathway result in its own inhibition [35]. The crosstalk between pathways is of significant interest in developing effective therapies. AKT activation was necessary in tumor cells’ progression in BRAF-mutated melanomas [36], despite its known inhibitory effect on RAF activity [37]. The release of cross-inhibition can increase signaling through an alternative pathway, as observed with MEK inhibition, leading to increased AKT activation [32]. Aside from pathway crosstalk, those pathways can be activated independently of RAS, which adds to the complexity of developing targeted therapies [38], as well as the differential behavior of mutant RAS isoforms in intracellular signaling [33].

Early attempts at targeting RAS focused on post-translational modifications to hinder its subcellular membrane trafficking. RAS prenylation through farnesylation of the CAAX motif of the c-terminus of RAS is the rate-limiting step in RAS trafficking to the cellular membrane [39]. Farnesyltransferase inhibitors (i.e., tipifarnib, lonafarnib, and others) showed efficacy in pre-clinical studies in HRAS-driven cancers, though failed to prove clinical efficacy in phase III clinical trials in patients with mutated KRAS [40]. KRAS and NRAS were found to have an alternative prenylation pathway for membrane localization via geranylgeranylation in comparison to HRAS, which was highly sensitive to FTIs [41]. This highlights that treating all RAS isoforms as one entity would be an oversimplification. Since prenylation is not unique to RAS proteins [42], selective targeting allows for greater precision and therapeutic selectivity. Though dual inhibition of Farnesyl and Geranylgeranyl Transferase were efficacious in pre-clinical research, such agents have not previously advanced to clinical testing [43]. A rejuvenated interest in FTIs in HRAS-mutated cancers is observed with trials underway (NCT04997902, NCT06026410), in addition to trials on novel Geranylgeranyl transferase I Inhibitor [44,45].

Given the complex pathway, simultaneous multiple forms of attack in the downstream effector pathway have been studied, demonstrating greater clinical benefit than targeting a single molecule. However, downstream regulators such as MEK, ERK, PIK3CA, and RAF have been difficult to target because of their roles in normal cell signaling [46]. Hence, given the challenges of targeting the downstream effectors themselves, current targeted treatments focus on blocking the process by which KRAS activates its downstream effectors. This provides a better therapeutic window, as targeting these processes enables the attack on cancer cells while sparing normal cells [47]. Several attempts have been made to identify small molecules that bind to RAS. The absence of deep hydrophobic pockets on the surface of K-Ras molecules makes it challenging to target this protein [48]. GTP binding has been considered undruggable due to its picomolar affinity. The absence of a suitable assay to detect small molecules that bind directly to RAS has limited progress in targeted drug development.

## 2. Emerging Strategies for RAS Targeting

### 2.1. Covalent Inhibitors (e.g., G12C Inhibitors, etc.), Their Chemistry, and Binding Modes

For about 40 years, there has been limited success in identifying drugs to inhibit RAS. The first covalent, mutant-selective inhibitor of K-RAS G12C was discovered in 2013. This class of inhibitors affects only the mutant protein, sparing wild-type KRAS. These covalent inhibitors selectively target the mutant cysteine residue in its GDP-bound inactive state in the pocket beneath switch 2 (S-IIP), thereby preventing K-RAS from converting to a GTP-bound conformation and further pausing downstream RAF and PI3K effector proteins. The first K-RAS G12C inhibitor with in vivo efficacy, called ARS1620, was discovered [49]. The rotation of His95 has revealed a cryptic groove adjacent to the switch II pocket [50]. A similar analog was later developed with the functional group extending around His95 without rotation of the residue. Sotorasib (AMG 510) and Adagrasib (MRTX849) each affects His95 in this druggable pocket [49]. These breakthroughs led to their clinical development as the first FDA-approved inhibitors of KRAS.

**Figure 3:**
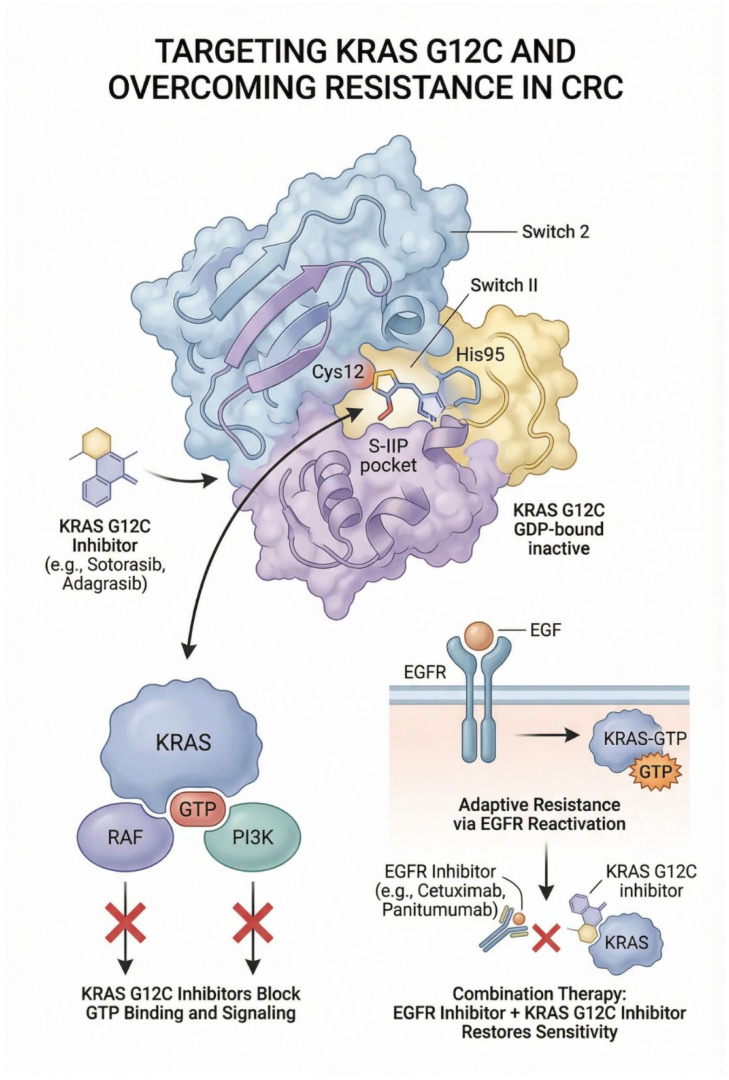
Mechanistic overview of KRAS G12C inhibition and combinatorial targeting with EGFR blockade in colorectal cancer (CRC). Description: This figure depicts the mechanism of KRAS G12C inhibition and resistance in colorectal cancer, showing covalent binding of KRAS G12C inhibitors to the switch II pocket of GDP-bound KRAS, thereby blocking downstream RAF and PI3K signaling. It also illustrates adaptive resistance via EGFR reactivation and highlights combination therapy with EGFR inhibitors to restore sensitivity to KRAS G12C–targeted treatment.

Despite the initial clinical efficacy of KRAS G12C covalent inhibitors as monotherapy, responses are often not durable. Particularly in colorectal cancer, adaptive resistance to KRAS G12C inhibition monotherapy has been observed via constitutive EGFR reactivation EGF stimulation decreases the potency of KRAS G12C inhibitors. In contrast, cotreatment with EGFR inhibitors such as erlotinib and afatinib decreases K-RAS GTP levels, thereby increasing K-RAS G12C inhibitor sensitivity [51]. Rational combinatorial blockade of EGFR reverts resistance to KRASG12C inhibition in CRC, which has translated into clinically effective regimens of Adagrasib and Cetuximab and Sotorasib and Panitumumab that have received regulatory approval [52,53] [Figure 3].

There are new oral KRAS G12C inhibitors that lock the protein in its inactive state, such as divarasib (GDC-6036). Compared to first-generation drugs, such as sotorasib and adagrasib, divarasib is 5–20 times more potent and 50 times more selective in vitro. A phase 1 study evaluated divarasib once daily in patients with advanced or solid cancers, among whom 3 patients had PDAC. In NSCLC, the results showed a response rate of 56.4% and a progression-free survival of 13.7 months. Among patients with colorectal cancer, the response rate was 35.9% and the median progression-free survival was 6.9 months [54,55]. Though the response rate in NSCLC and colorectal cancer was significant, more studies with divarasib in PDAC are necessary for quantifying its efficacy in this population.

One drug candidate, BBO-8520, has been shown to target both the GDP-bound and GTP-bound states of KRAS G12-C successfully. Another such drug, which binds to the ON-state of KRAS, is RMC-6291. This drug is known to form a tricomplex with peptidylprolyl isomerase A (PPIA), a cellular chaperone protein that stabilizes drug binding to the active, GTP-loaded conformation of KRAS [56,57].

### 2.2. Novel Inhibitors for Non-G12C Mutants (G12D, G12V, Etc.)—Recent Preclinical Developments

Other KRAS inhibitors has focused on G12D and G12V, which are among the most common KRAS mutations observed. KRAS G12D and KRAS G12V make up to 39% and 31% of KRAS mutations in PDAC, respectively. Depending on the type of KRAS mutation, prognosis widely varies. KRAS G12D mutations correlate with poor prognosis [52]. A recent development in this area includes Mirati’s MRTX 1133, the first non-covalent KRASG12D inhibitor. The mechanism of action of MRTX113 includes binding to GDP-bound inactive KRAS G12D and inhibiting the binding of RAF-RAS binding domain peptide to the active form of KRAS with 700× selectivity. Data from preclinical studies suggest that inhibitors such as MRTX1133 could usher in a new era in the treatment of PDAC [53]. However, clinical trials with this candidate drug have been terminated due to unfavorable pharmacokinetics. There are various other small molecule KRAS G12 D inhibitors under clinical evaluation including GFH375/VS-7375 (NCT06500676), HRS-4642 (NCT05533463), INCB161734 (NCT06179160), LY3962673 (NCT06586515), TSN1611 (NCT06385925), QLC1101 (NCT06403735), and QTX3046 (NCT06428500) [58,59] [Table 1].

A covalent inhibitor of KRAS G12D has been designed by binding the abundant intracellular chaperon cyclophilin A (CYPA), forming a neomorphic protein-protein interface between CYPA and active RAS. This interface further modifies the D12 mutation located in the induced pocket at the interface. The investigation agent was named Zoldonrasib (RMC-9805). This drug has been further investigated in clinical trials across xenograft models of KRAS G12D mutant cancer. This drug has also been investigated in 9 patients with PDAC. This study showed that oral administration of Zoldonrasib at 100 mg/kg was well tolerated and that tumor regression was observed when it was combined with a MAPK pathway inhibitor. This drug is currently in clinical evaluation (NCT06040541) [60].

Although no targeted G12V inhibitor has been described to date, some preclinical data of KRAS G12V inhibitors that promote a tri complex between the inhibitor, active GTP bound state of KRAS G12V and cyclopholin A (Cyp A). The above mutation may need more studies to determine its role in developing targeted treatments for PDAC, particularly for RAS-addicted cancers [61].

### 2.3. Protein Degraders

A newly emerging approach is proteasome-targeting chimeras, which result in specific post-translational degradation of the target protein. It comprises bifunctional small molecules containing a ligand and a specific target of interest. This is further coupled to a ligand for a particular E3 ubiquitin ligase complex. PROTAC uses this mechanism to recruit the Ubiquitin ligase machinery to target a protein for polyubiquitination and subsequent proteasomal degradation. PROTAC has a specific advantage over knockdown methods, as it can be effective against proteins with longer half-lives. Another crucial feature of PROTAC is its mechanism of action, which is event-driven rather than occupancy-driven in traditional pharmacology [62].

Peptide PROTACs, also known as peptide degraders, offer an alternative approach to small-molecule PROTACs. These are small-molecule heterobifunctional degraders comprising three components: an E3 ubiquitin ligase, a high-affinity binder specific for the target of interest, and a linker that joins the two protein domains. There are 25 degrader drugs currently in clinical trials for multiple indications. The main advantage of peptide-PROTAC is that it can be recycled to initiate multiple rounds of RAS degradation. Ma et al. effectively applied the peptide-PROTAC to inhibit KRAS. Treatment with the proteasome inhibitor MG-132 rescued KRAS expression, demonstrating proteasome-dependent regulation in RC-U-transfected PANC-1 cells. This showed proteasomal targeting of KRAS was possible using a peptide-PROTAC approach, and it suppresses pancreatic cancer cell growth in vitro and in vivo [63,64].

The one crucial KRAS degrader in clinical trial as of April 2025 is ASP3082 (NCT05382559). This quinolone-based KRAS G12D binder had two linker sites. Exit a is used in previously reported KRAS G12C degraders, while exit b was unique to the KRAS binder in the study. This ternary complex-based optimization led to the potent discovery of KRAS G12D degrader ASP3082 [65]. In another study, the safety and efficacy of RP03707, a highly selective and potent KRAS G12D inhibitor, were evaluated. Its potent activity demonstrated superior antitumor activity in pancreatic cancers in vivo [66]. In a recent study, CH091138 was identified as a selective degrader of both exogenous and endogenous KRAS G12D, with greater selectivity over KRAS WT and other KRAS mutants, including KRAS G12C, G12S, and G12V [67].

### 2.4. Macrocyclic Peptides and Pan KRAS and RAS Inhibitors

Through advanced techniques such as structure-guided screening, molecules with nanomolar affinity have been developed. One such molecule is the macrocyclic scaffold, which binds to the Switch I/II pocket of GDP-bound KRAS-G12D [68]. The interaction between KRAS and SOS1 is effectively blocked by the compound KRpep-2d, thereby preventing RAF-MEK-ERK activation and downregulating pERK signaling in KRAS G12D mutant cells [69]. This development has enabled the manufacture of potential derivatives that, by disrupting RAS-effector interactions, have led to activity against KRAS-G12D and KRAS-G12V mutants. In preclinical trials, these peptides have been shown to play an important role in tumor regression in this mutant cell population [70].

In preclinical models, BI-2852 suppresses growth across KRAS G12C, G12D, and G12V by binding to the inactive state, whereas BI-2865 is a pan-KRAS inhibitor, a metabolically stable, permeable structural analog of BI-2852. Another interesting pan-KRAS inhibitor is MCB-294, which also targets multiple mutants by targeting dual KRAS states [71]. BI-3706674 (NCT06056024) is an oral small-molecule KRAS Inhibitor. This oral regimen is on a phase 1 clinical trial testing the investigational drug BI-3706674 in adults with advanced or metastatic cancer of the esophagus, gastroesophageal Junction, or stomach [72]. LY-4066434 (Eli Lilly) is a pan-KRAS inhibitor currently in a phase 1a/1b study of patients with locally advanced or metastatic solid tumors harboring KRAS mutations. The notable feature of this drug is that, in preclinical models, it has demonstrated CNS penetration [73,74]. Lastly, PF-07934040 (Pfizer) is another phase 1 open-label study worth mentioning. This drug is also a pan-KRAS inhibitor, a small-molecule agent that blocks multiple KRAS mutant forms [75]. In the current literature, there are two studies on RMC 6236 and three on RMC 7977, both of which are multi-selective RAS(ON) inhibitors. RMC 7977 demonstrated two critical outcomes: first, significant antitumor activity; and second, a significant prolongation of time to tumor doubling. RMC 6236 in phase 1 clinical trial elicited an objective response rate (ORR) of 20% and a disease control rate of 87% in PDAC. Both drugs showed concentration-dependent inhibition of the pathway. Pan-RAS inhibitors are also emerging as an experimental drug in ovarian cancer. As noted, multiple trials are ongoing across the spectrum of KRAS and RAS inhibitors, and additional data are expected to be published in the next 1–2 years [76,77,78,79].

### 2.5. Allosteric Modulators & Switch Region Targeting

The KRAS active site comprises the phosphate-binding loop (P-loop), switch I, and switch II regions, and its conformation is required for KRAS activation. In its GTP-bound state, K-RAS can adopt either an open or a closed conformation. The conformational changes undergone by Switch I and Switch II are essential for binding the effector protein. The state I conformation is incompatible with effector protein binding. In contrast, in state II, high-affinity binding to effector protein such as RAF1 is facilitated by switch I adopting a closed conformation [80]. Oncogenic mutations in the RAS protein at residues Q61, G12, and G13 lock RAS in its active, GTP-bound conformation by impairing GTP hydrolysis [81].

The two proteins, GTPase-activating proteins (GAPs) and guanine nucleotide exchange factors (GEFs) play a crucial role in regulating KRAS protein. The disruption of these proteins can alter regulatory mechanisms, leading to aberrant signaling and disease progression. Hence, these sites are potential targets for therapeutic interventions [82].

The allosteric pockets are amenable to small-molecule binding, especially the switch II pocket, which has attracted attention as a potential drug target. Drugs targeting this site can act through two mechanisms: either stabilize the inactive state of KRAS or disrupt protein–protein interactions. The interaction between KRAS and GEF has been identified as another potential target [83].

### 2.6. Blocking Pathway Crosstalk

With pathway inhibitors (e.g., MEK, ERK, PI3K), immunotherapy, chemotherapy, etc. Other important pathway inhibitors include PI3K and MAPK pathways, which are potential downstream targets in mutant KRAS. FDA approval of MEK1/2 (MAPK pathway) called Trametinib has already been obtained for colorectal cancer. This drug is used in combination with the BRAF inhibitor dabrafenib for all non-resectable or metastatic solid tumors (except colorectal cancer). Various clinical trials have tested the efficacy of Trametinib in pancreatic cancer but have demonstrated limited efficacy, likely due to resistance [84]. To overcome this resistance, many recent studies have focused on combination therapies that block the reactivation pathways. The PI3K and MTORC 1/2 inhibitor Omipalisib has demonstrated moderate efficacy in clinical trials for solid tumors. Combined MEK and PI3K inhibition have shown efficacy in PDAC, but it has also faced challenges due to therapeutic toxicities [85].

Combining CDK4/6 inhibitors with ERK-MAPK inhibitors in PDAC may have a synergetic effect. When used in tandem lower concentrations of both ERK 1/2- selective inhibitors (ERKi) SCH772984 and CDK4/6i were required to cause loss of proliferation than when used separately. Combining these therapies sensitized PDAC lines, which were initially resistant to CDK4/6i. Recent data have also demonstrated that combining CDK4/6i with KRAS G 12C enhances its effect [86]. Similarly, gemcitabine combined with the MEK inhibitor trametinib showed an enhanced antitumor effect compared with gemcitabine alone. Moreover, in gemcitabine-resistant PDAC, the combination of the MEK inhibitors trametinib and cobimetinib prevented tumor growth in mouse models [87].

### 2.7. Synthetic Lethal Approaches

Synthetic lethality can be triggered when two genes in the same signaling pathway harbor loss-of-function mutations, when two genes activate the same pathway through distinct signaling cascades, or when genes targeting different cellular pathways converge to achieve a specific cellular outcome. KRAS-mutant cells may harbor specific genetic alterations that are amenable to therapeutic targeting [88]. These vulnerabilities arise from oncogenic activation, and mutant KRAS induces an overall stress state encompassing mitotic, metabolic, proteotoxic, oxidative, and replicative stress. In addition, genetic backgrounds further hinder the identification of synthetic lethal interactions. These factors explain why an effective therapy based on synthetic lethality has not proved to be clinically effective [89].

Genes including PLK1, TBK1, STK33, YAP1, FGFR1, WT1, and XPO1, have been implicated in synthetic lethal interactions with KRAS. Several clinical trials have evaluated the efficacy of KRAS inhibitors, including CYC140 (Phase I: NCT03884829) and BI-2536 (Phase II: NCT00710710), which are anti-cancer drugs designed to target the enzyme PLK1, which plays a crucial role in cell division [90].

There has been pioneering work in identifying synthetically lethal genes using CRISPR/Cas9 screens in the context of oncogenic KRAS. One study found that RAS-targeted therapy acts on specific components of the RAS pathway, thereby affecting the viability of RAS-dependent tumor cells in AML cell lines. Modifications in RAF1 and SHOC2 genes have supported the role of MAPK signaling in RAS-mutant cancers [91].

### 2.8. Upstream Pathway Inhibition

Two mechanisms of resistance to G12C GDP inhibitors are increased upstream signaling and increased KRAS expression and amplification [92]. This signaling further generates active SOS, which competes with GDP-state inhibitors for binding. SOS competes with KRAS G12C for binding and cycles it to the GTP state, thereby preventing the action of KRAS G12C drugs, which predominantly bind to the GDP-bound state of KRAS. This, in turn, leads to increased GTP-bound KRAS production, which is drug resistant. A fraction of KRAS G12C can increase protein expression by escaping drug binding [93].

Recent data have also suggested that the tyrosine phosphatase SHP2 is an essential factor in RAS-mediated MAPK signaling and is required for tumorigenesis in mutant KRAS-driven PDAC. Combining SHP2 with either MAPK or PI3K inhibitors has shown efficacy in multiple cancer types, but their use in PDAC remains under investigation [94].

An SOS-1 Inhibitor works by blocking interactions between SOS1 and RAS-GDP. One such orally available and highly potent drug is BI3409. This drug has proven that the SOS1-KRAS interface is a clinically crucial druggable target [95]. Combining therapies is essential to improve KRAS G12C inhibition in both colorectal and NSCLC. KRAS G12C binds only to the inactive GDP-bound state. TNO155 is an allosteric inhibitor of tyrosine phosphatase SHP2 (encoded by PTPN11), which facilitates this by reducing GTP loading, thereby increasing the KRAS G12C GDP-bound state. In addition to this, TNO155 also prevents pathway activation mediated through HRAS, NRAS, and (WT) KRAS, which the KRAS G12C Inhibitor cannot target [95,96].

### 2.9. RNA-Based Therapies (siRNA, Antisense, RNA Interference)

It remains a therapeutic challenge to target non-G12C oncoproteins, such as KRAS, using siRNA. The development of an anti-cancer vaccine targeting mutant KRAS and adoptive T cell therapies targeting mutant KRAS are also key areas of focus [97].

Silenseed designed LODER TM. This is a miniature Biodegradable matrix that enables slow, prolonged local release of the encapsulated drug. Exosome-based delivery of KRASG12D siRNA (iExoKRASG12D) achieves pancreas localization with benign safety in nonhuman primates and shows feasibility in a Phase I trial, supporting biologically “stealth” carriers for hard-to-reach PDAC sites [98]. Local depot approaches such as siG12D-LODERT sustain intratumoral siRNA release, demonstrating a preliminary survival benefit when combined with chemotherapy in early-phase studies [99,100]. A phase 1 study is evaluating the optimal dose of mesenchymal stromal cell-derived exosomes containing KRAS G12D siRNA for the treatment of pancreatic cancer patients with KRAS G12D mutations and metastatic disease [101].

A molecule simultaneously inhibiting KRAS and MYC has been investigated. This is an “inverted” RNAi molecule in which a MYC-targeting siRNA strand is fused to a guide KRAS-targeting siRNA. Furthermore, improved metabolic stability with decreased tumor progression was observed when combining siRNA and EGFR-targeting ligands [102].

Some preclinical studies in mice and rhesus macaques have shown negligible toxicity in pancreatic cancer when engineered exosomes expressing KrasG12D-specific siRNA (iExoKrasG12D) were administered. This drug, when combined with anti-CTLA4, showed a significant tumor response, which was due to a unique mechanism of immune microenvironment remodeling by oncogenic KRAS suppression following iExoKrasG12D treatment [98].

Furthermore, a preclinical evaluation of a high-affinity, ethyl-constrained therapeutic antisense oligonucleotide (ASO) called AZD4785 has been conducted. Delivery of this oligonucleotide to mice bearing KRAS-mutant NSCLC patient-derived xenografts resulted in inhibition of KRAS expression in tumors and a favorable safety profile [103].

### 2.10. Immune Targeting

Recent studies have demonstrated that anti-PD-1 therapy with KRAS inhibition enhanced antigen presentation and cross-presentation in genetically engineered mouse model IKPC PDAC. This is due to improved priming and activation of tumor-specific T-cell responses, which leads to effective anti-tumor activity [104]. Conventional type 1 dendritic cells (cDC1s) enhance cross-presentation in PDAC, and costimulatory 41BB signaling is essential for sustaining the survival of activated dendritic cells. Combining MRTX1133 treatment with triple IO led to better efficacy, which may be attributed to the agnostic anti-41BB antibody [104,105].

Adjuvant autogene cevumeran, an individualized mRNA neoantigen vaccine, in combination with atezolizumab and mFOLFIRINOX, has been shown to induce and enhance T-cell activity, which may play an essential role in eliminating micrometastases in surgically resected patients with PDAC. Furthermore, Cancer vaccines underscore the importance of antigen presentation enhancement by modulating the T-cell progenitor population and may represent a future therapy in surgically resected PDAC [106].

Tumors harbor neo-antigens that can be targeted to stimulate an immune response against cancer cells. These vaccines use mRNA encoding a mutant KRAS, encapsulated in a nanoparticle that enhances the immune response against tumor-associated neoantigens. mRNA-5671/V941 encodes a mutant KRAS and is currently in a phase 1 clinical trial investigating it in KRAS-mutant tumors (NCT03948763). Clinical trials are investigating vaccines as single agents and in combination with the PD-1 antibody Pembrolizumab. Various other vaccines, including dendritic cell vaccines (NCT03592888) and peptide vaccines (NCT04117087), are in clinical trials in KRAS-mutated patients [107].

## 3. Clinical Development & Trials in Pancreatic Cancer

Various clinical trials are ongoing to target the KRAS mutation in pancreatic ductal adenocarcinoma. There have been significant efforts made in the pan-KRAS inhibitor front. The following studies were instrumental in the emergence of KRAS inhibitors, including BI-3706674, LUNA18, LY4066434, and PF-07934040. These molecules bind to the inactive state of KRAS (KRAS(OFF)) and to a groove between switch I and switch II regions of the KRAS protein [108].

Various recent studies by Revolution Medicine for the RAS (ON) protein have been designed. To date, RMC-7977, a noncovalent reversible pan-RAS (ON) inhibitor, has been successfully identified. The mechanism of action is a tri-complex inhibitor against both mutant and WT KRAS, NRAS, and HRAS variants. Two irreversible RAS G12C (ON) Inhibitors have been discovered: RMC 4998 and elironrasib (RMC-6291) [78,108].

Daraxonrasib binds to the composite binding pocket of the RAS (ON) protein. In earlier phase trials, it has proven to be an effective tricomplex multi-selective inhibitor similar to RMC-7977; in addition, it overcame resistance to first-generation KRAS G12C (OFF) Inhibitors. Hence, Daraxonrasib is superior to selective KRAS G12C inhibitors targeting KRAS G12C, G12D, and G12V [108]. One of the global randomized, open-label phase 3 multicenter trial in patients already treated for metastatic PDAC. This study compared Daraxonrasib (RMC-6236) with the investigator’s choice of standard-of-care therapy (RASolute 302), which is ongoing but closed to accrual and includes documented RAS mutation status, either mutant or wild-type. In this study, RAS mutations are defined as nonsynonymous mutations in KRAS, NRAS, or HRAS at codons 12, 13, or 61 (G12, G13, or Q61) [109].

A Phase 1/2a study of IMM-6-415 in participants with advanced or metastatic malignancies harboring RAS or RAF oncogenic mutations included pancreatic ductal carcinoma in the study. This study included participants who had previously received one line of systemic standard-of-care treatment for their advanced or metastatic disease. Additionally, the study included KRAS G12C-mutant participants who received KRAS G12C inhibitors for any approved indication. The study’s goal is to determine whether IMM-6-415 has a safe dose at which tumor shrinkage and disease progression can be slowed. The result of the study is yet to be published [110].

Other phase 2 clinical trials include a study to evaluate ARV-806 in adults with KRAS G12D mutation and advanced disease [111]. Another trial is a first-in-human Phase 1/2 Trial of ELI-002 7P Immunotherapy as a treatment for subjects with KRAS/NRAS-mutated pancreatic ductal adenocarcinoma (PDAC) and other solid tumors [112]. A study of RAS (ON) inhibitors in patients with gastrointestinal solid tumors. The Subprotocol F in this study is an open-label multicenter study of RMC-9805 with or without RMC-6236 in combination with gemcitabine and nab-paclitaxel in patients with RAS G12D-mutant metastatic pancreatic ductal adenocarcinoma [113]. Another ongoing study is evaluating Avutometinib (VS-6766) and Defactinib in combination with chemotherapy such as gemcitabine and nab-paclitaxel in patients with previously untreated metastatic pancreatic adenocarcinoma [114]. One other clinical trial in this spectrum includes a study of agents targeting the mitogen-activated protein kinase pathway in patients with advanced gastrointestinal malignancies (HERKULES-3). This trial has the following inclusion criteria, which include patients who have histologically or cytologically confirmed metastatic CRC harboring applicable mutation(s) (e.g., BRAF V600E; KRAS or NRAS mutations) or metastatic PDAC harboring KRAS mutation based on an analytically validated assay performed on tumor tissue in a certified testing laboratory [115]. The analysis and results of many of these trials are awaited [Table 1].

**Figure 4:**
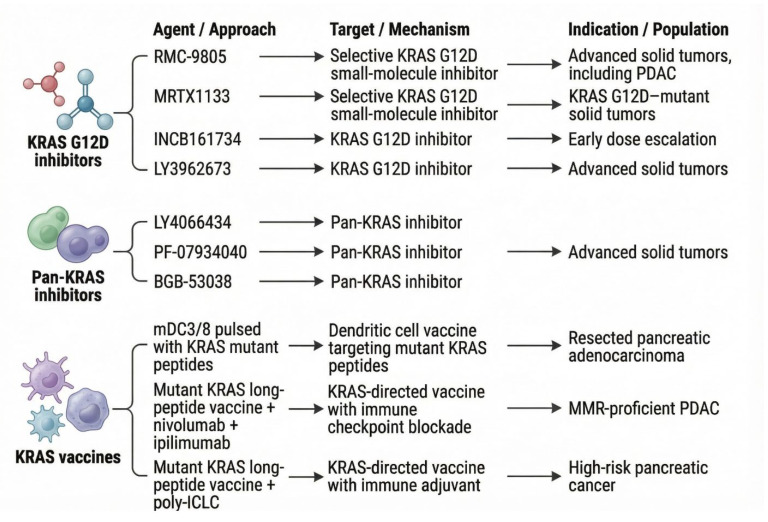
Overview of selected Phase I clinical trials relevant to pancreatic ductal adenocarcinoma (PDAC). Description: This figure depicts the selected phase 1 clinical trials in the context of KRAS G12D inhibitors, Pan-KRAS inhibitors, and KRAS vaccines. The agent’s name, approach, mechanism of action, and the type of cancer included in the trial have been summarized.

The following drugs, including RMC-9805, MRTX1133, INCB161734, LY3962673, are in Phase 1 clinical trials of G12D inhibitors [73,110,116,117]. Various early-phase trials are investigating Pan-KRAS inhibitors, including LY4066434, PF-07934040, and BGB-53038 [118]. Furthermore, there are various KRAS vaccine phase 1 trials which include a study where mature dendritic cells (mDC3/8) are “pulsed” with KRAS mutant peptides and administered to patients with resected pancreatic adenocarcinoma [119]. Another trial investigates a pooled mutant KRAS-targeted long peptide vaccine in combination with nivolumab and ipilimumab in patients with mismatch-repair-proficient PDAC [120]. Another Phase 1 study investigates a mutant KRAS-targeted long-peptide vaccine, combined with adjuvant poly-ICLC, in patients with high-risk pancreatic cancer [121] [Figure 4]. The clinical trials included in this review do not encompass all ongoing trials in PDAC; we have included only those most relevant to our review.

## 4. Immune Cold Microenvironment

Preclinical trials have attempted to reverse the cold tumor microenvironment. One Phase-1 study demonstrated that CD8+ T cells are required for sustained suppression of tumor growth and clearance. They explained the critical role of CD8+ T cells in tumor clearance when MRTX1133 was initiated in mice with a large tumor burden and advanced PDAC [122]. MRTX1133 reprogrammed the tumor microenvironment by blocking KRAS G12D and led to depletion of Myeloid-derived suppressor cells (MDSCs) that secrete higher levels of immunosuppressive cytokines, such as IL-10 and TGF-β, thereby reducing the number of natural killer T cells. The accumulation of MDSCs has been seen in KPC mouse models of PDAC with a negative correlation with CD8+ T cell infiltration. Thus, MRTX1133 had three main effects on the tumor microenvironment, which include reprogramming cancer-associated fibroblasts, increasing infiltration of CD8+ effector T-cells, and decreasing myeloid cell infiltration [123].

KRAS codon-specific alterations affect TME composition. In a retrospective, multicenter, observational cohort analysis, the TME of PDAC was compared between KRAS G12R and G12D tumors, showing that G12R tumors exhibited much lower PD-L1, inflammatory cytokine, and inhibitory checkpoint expression, resulting in a lower T cell-inflamed signature score and neutrophil count in G12R than in G12D. The high immune resistance seen in PDAC prevents monoclonal antibodies (mAbs) from targeting either the CTLA-4 (cytotoxic T-lymphocyte-associated protein 4) or PD-1 (programmed cell death protein 1) axes. Notably, the G12R subgroup maintained the highest overall survival among all codon 12 variants, despite a low immunogenic response [124].

### 4.1. Challenges Associated with Targeting Different Amino Acid Substitutions in Codon 12 of KRAS

KRAS-mutated cancers harbor distinct mutation subtypes (and can harbor co-mutations), making them heterogeneous. For example, KRAS Q61 mutations impair GTP hydrolysis, leading to persistent KRAS activation and downstream signaling. KRAS G12D harbors an intrinsic wild-type and SOS1 guanine exchange activity; G12D was also reported to be more immunosuppressive, leading to overall shorter survival in lung and pancreatic cancers. The KRAS G12R mutation (which accounts for 15% of KRAS mutations in pancreatic cancer but less than 1% in lung cancer) is associated with distinct downstream signaling compared to other KRAS subtypes. One institutional study compared overall survival (OS) between G12R and G12D mutations and found that the G12D group had worse OS. However, within the G12R group, a co-mutation involving the PI3K pathway was associated with lower OS. Other studies have found no statistically significant difference in OS across allele subtypes, indicating the need for further investigation [125].

Another dataset showed KRAS G12R tumors to have the highest incidence of co-mutations within the suppressor genes (SMAD4, TP53, and CKDN2A), which could help explain its rarity (13% incidence) compared to G12D (49%) and G12V (23%) [126,127]. KRAS G12R also directly upregulated PI3K activity, a finding not observed with the G12D or G12V variants. This defective signal in G12R may also affect PDAC’s resistance to KRAS inhibitors, potentially necessitating alternative therapeutic approaches [127].

### 4.2. Resistance Mechanisms: Adaptive Signaling, Feedback, Bypass Tracks

Primary resistance is defined as a failure of cancer cells to respond to KRAS inhibitors at the initiation of treatment. In this population, Co-occurring mutations in tumor suppressor genes strongly shape therapeutic response. Resistance mechanisms remain a prominent barrier to having consistent success with KRAS-targeted therapy in PDAC.

Upstream resistance:

Recent work has also demonstrated the importance of EGFR and other ERBB family members in mediating adaptive responses and resistance to KRAS inhibition [128]. There are diverse molecular and cellular mechanisms by which KRAS-mutant PDAC exhibits intrinsic or adaptive resistance, resulting in transient and inconsistent clinical responses to targeted therapies. In one of the studies, it was demonstrated that colorectal cancer (CRC) patients who are treated with KRAS G12C inhibitor monotherapy benefit less than NSCLC patients. Interestingly, CRC patients treated with the KRAS G12C inhibitor were more responsive to growth factor stimulation, as they exhibited higher basal tyrosine kinase receptor activation. This mechanism was responsible for resistance to KRAS G12C inhibitor monotherapy. Hence, these studies determined that KRAS G12C inhibitors combined with anti-EGFR agents are a plausible option in CRC [129]. In addition, KRAS G12C has been studied in the context of mutations such as KEAP1, STK11, and elevated PDL1 expression, which are associated with poorer prognosis due to primary resistance [130].

Downstream resistance:

One of these methods involves adaptive signaling, the rapid “reprogramming” of signal pathways in response to pressure from specific therapies. For example, inhibiting downstream effectors of KRAS (MEK, AKT, etc.) can lead to a compensatory increase in alternative receptor tyrosine kinases (RTKs), such as the ERBB family, thereby restoring MAPK and PI3K activity. This is evident by the rise in ERBB2/ERBB3 phosphorylation following MEK/AKT downregulation [131]. This is also an example of resistance through feedback loops, as negative feedback from the MAPK signaling pathway halts the suppression of upstream RTKs, leading to their hyperactivation and eventual restoration of downstream signaling.

Bypass track resistance:

Bypass tracks are also a prominent mechanism in PDAC that promotes resistance, in which tumor cells circumvent blocked signaling pathways and activate lesser-used alternative routes to promote growth. Studies have shown that PDAC cells activate “escape” routes, such as the YAP1, TGF-Ꞵ, or NOTCH pathways [132,133].

KRAS G12C inhibitor resistance can be overcome by targeting the SRC-JUN pathway. Here, SRC, via RAF1-MEK-ERK, can lead to JUN activation. This activation can lead to JUN phosphorylation [134]. IAG933 is an experimental oral drug that works by blocking TAP/TAZ-TEAD. Inhibition of this protein interaction is an effective pharmacological strategy that improves the response to KRAS G12C inhibitors [135]. These bypass routes enable PDAC to maintain tumor growth despite KRAS suppression, thereby conferring resistance.

Acquired Resistance:

Genetic resistance mechanisms have also been acquired, specifically against KRAS G12C inhibitors. An analysis by Riedl et al. found invariable resistance to selective KRAS G12C inhibitors, despite their initial and short-lived efficacy against G12C cancers. Patients were treated with either a single KRAS G12C inhibitor agent or a combination of anti-EGFR antibodies. Results showed that RAS/MAPK alterations developed in 46% of patients, with 39% acquiring at least one new KRAS alteration and 23% exhibiting multiple concurrent alterations. The genomic picture of alterations was diverse, including KRAS-activating mutations, KRAS amplifications, RAF/MAPK mutations, KRAS switch-II pocket mutations, and NRAS/HRAS mutations; functional studies also confirmed that most of these changes were indeed drivers of resistance. It is essential to note that the study focused heavily on colorectal cancer (CRC) and non-small-cell lung cancer (NSCLC) rather than PDAC [136,137,138]. KRAS alterations can lead to resistance. There have been mutations detected in the switch II pocket, such as H95D/G/N/R, Y96C/D/H/N, and R68S, that restore KRAS activity by reducing inhibitor affinity and disrupting drug binding [139].

Allele-specific resistance:

Adaptive resistance has also been observed around the wild-type (WT) KRAS allele, which usually acts as a tumor suppressor when mutated. As the tumor progresses, it can either lose the WT allele or acquire additional copies of the mutant allele; tumors that exhibit either a gain of the mutant allele or a loss of the WT allele are expected to have a worse prognosis than patients with balanced KRAS mutations. However, only the mutant allele dosage was a significant predictor of prognosis, regardless of whether the WT allele was lost or retained. Whether these alterations are acquired (as seen with TP53 or SMAD4) or germline, resistance to RAS therapeutics remains a significant challenge when assessing the evolving field of KRAS targeting [1,140].

## 5. Future Perspectives & Emerging Opportunities

### 5.1. Novel Targets in the RAS Pathway, or in Parallel Pathways

Beyond mutant KRAS itself, multiple actionable upstream and downstream nodes have emerged, including RTKs (EGFR/ERBB family), SHP2 (PTPN11), and SOS1, which can be co-targeted with KRAS inhibition to blunt feedback reactivation of MAPK signaling and improve durability of response [95,141,142,143,144,145,146]. Pan-ERBB blockade potentiates anti-KRASG12D therapy in preclinical PDAC, consistent with clinical experience in EGFR-addicted malignancies and highlighting ERBB-axis coactivation as a common bypass route [112,141]. It has been shown in Phase 1 clinical trials that HER2 activation provoked by MAPK pathway inhibition can be neutralized using an anti-HER2 ADC in combination with ERK/MAPK inhibitors, enabling sustained tumor regressions in PDAC patient derived xenografts (PDXs) and motivating dual-pathway strategies that intercept adaptive receptor-level signaling [147]. Parallel stress- and cell-cycle nodes are tractable combination partners; KRAS inhibitors synergize with CDK4 blockade to suppress PDAC growth across KRAS variants, and PLK1 inhibition enhances effects of MAPK-targeted therapy, underscoring mitotic/cell-cycle co-targeting as a viable approach [86,147,148]. Furthermore, novel mechanistic insights into KRAS-dependent nuclear protein export implicate DLC1 as a tumor suppressor target; combined inhibition of KRAS with AKT and SRC kinases cooperatively restores DLC1 function and amplifies antitumor activity in PDAC models [26,149]. The studies described above are in phase 1 clinical trials, and further evidence is needed to establish their safety and efficacy in human subjects.

### 5.2. Biomarker-Driven Clinical Trial Design

Prospective platform and basket trials (e.g., KRYSTAL-1 for G12C; emerging RASolute 302 for pan-RAS(ON) inhibitor RMC-6236) underscore adaptive designs that match drugs to genotypes (G12C, G12D/V/R) and refine combinations based on on-treatment ctDNA and resistance landscapes [109,150,151]. Real-world and trial data indicate meaningful activity of G12C inhibitors in PDAC (ORR ~21–33%) despite low prevalence, validating the principle of targeting KRAS [137,146,152].

Comprehensive resistance mapping across inhibitor classes (mutation-selective OFF-state vs. pan-RAS(ON)) reveals recurrent alterations in RAS/MAPK, RTKs, PI3K, and MYC; biomarker-informed combinations (e.g., ERBB blockade, SHP2/SOS1 inhibition, TEAD co-targeting) should be prospectively embedded to pre-empt relapse [137,146,150,153]. Variant-informed precision (allele subtype and copy number dosage) displays differences: KRASG12D associates with worse outcomes than G12R and distinct metabolic/MAPK wiring; mutant allele dosage and LOH correlate with survival and may modulate sensitivity to MEK and RAS inhibitors, supporting stratified enrollment and tailored regimens [100,124,154,155].

KRAS wild-type PDACs harbor a richer set of actionable targets (e.g., RTK fusions, BRAF alterations) and improved outcomes, reinforcing upfront broad profiling (tissue and ctDNA) to capture trial eligibility (ESCAT tiers) and to direct patients to appropriate targeted combinations [128,156]. Epidemiologic and genomic baselines (RAS mutation frequencies, incidence estimates, co-mutation patterns) support rational accrual planning and underscore the need for global biomarker infrastructure to conduct adaptive trials efficiently in PDAC [157,158].

Finally, upstream and downstream pathway targeting remains central in PDAC given KRAS dominance and frequent TP53/CDKN2A/SMAD4 alterations; vertical inhibition strategies (RTK → RAS → MAPK), and modulation of parallel metabolic and cell-state programs are crucial to overcome multifactorial resistance [132,159]. The extracellular niche considerably influences KRAS inhibitor efficacy; accounting for stromal architecture and metabolic rewiring (e.g., lipophagy–FAO vulnerabilities) can inform biomarker selection and rational combinations in the clinic [122,148,160]. Preclinical studies and translational reviews emphasize that robust responses will require multi-pronged approaches, combining next-generation KRAS modalities with IO, RTK/TEAD/SHP2/SOS1 co-inhibition, apoptosis/senolytic strategies, and optimized delivery, implemented through adaptive, biomarker-led trials [48,79,161,162,163].

### 5.3. Integration with Immunotherapy, Targeting the Microenvironment

KRAS inhibition reshapes PDAC’s immunosuppressive milieu: RAS(ON) multi-selective inhibitors reduce myeloid dominance and increase T-cell infiltration; when combined with PD-1, CTLA-4, or CD40 agonists, they deepen responses and yield complete regressions in immunocompetent models [164]. Allele-specific G12D inhibition (MRTX1133) upregulates FAS, a cell-surface death receptor and primes CD8+ cytotoxicity, restoring immune surveillance in early and advanced diseases, and supporting combination with immune checkpoint blockers to eradicate tumors and prolong survival [122].

A multi-component immunotherapy (IO) regimen (CXCR1/2 inhibitor + anti-LAG3 + anti-4-1BB) layered on KRAS inhibition generated durable complete responses in autochthonous PDAC, underscoring the need to address myeloid suppression, T-cell exhaustion, and DC cross-presentation concurrently [104]. Active-state inhibition (RMC-7977) exhibits tumor-selective apoptosis with minimal normal-tissue cytotoxicity, suggesting a therapeutic window for pairing RAS blockade with IO, especially in the setting of TEAD/YAP/MYC-driven resistance that may blunt immune effects [165].

### 5.4. Vaccinations and Tumor Microenvironment Targeting

Vaccination strategies and adoptive cellular therapies are maturing. KRAS-specific amphiphile vaccines (ELI-002) elicit robust T-cell responses and ctDNA clearance in PDAC MRD, whereas TCR therapies targeting KRAS neoepitopes have produced clinical regressions, providing immunologic complements to direct RAS targeting [106,166,167]. Targeting adaptive checkpoints in the TME can potentiate KRAS inhibition; for example, CD24 is upregulated upon KRASG12C inhibition, and co-blockade enhances macrophage phagocytosis and sensitizes tumors to RAS therapy [1,168,169].

### 5.5. Macropinocytosis

In a nutritionally poor environment, PDAC relies on macropinocytosis to sustain anabolic metabolism. Oncogenic KRAS drives this mechanism [170]. KRAS G12D inhibitors suppress macropinocytosis via inhibition of Rac family small GTPase 1(RAC 1) in MRTX1133- sensitive pancreatic ductal adenocarcinoma. The activation of RAC1 that occurs via β-catenin and advanced glycosylation end-product specific receptor (AGER, also known as RAGE)–diaphanous related formin 1 [171]. Macropinocytosis suppresses apoptosis by promoting amino acid uptake and glutathione synthesis, thereby reducing cell death. Similarly, adaptive resistance to G12C inhibitors is mediated by AGER-dependent mechanisms in NSCLC [88]. Hence, targeting AGER-driven macropinocytosis improves the efficacy of KRAS inhibitors in PDAC by overcoming metabolic resistance [172].

### 5.6. Autophagy

Autophagy is a well-known pathway that involves lysosomal degradation of organelles, cellular components, and misfolded proteins, leading to drug resistance [173]. MRTX1133 can induce autophagy through mTOR suppression in KRAS-G12D-mutant PDAC. This leads to enhanced glutathione synthesis, inhibition of cytochrome c-mediated apoptosis, and decreased reactive oxygen species accumulation. Genetic deletion of autophagy-related 5 or beclin 1, as well as pharmacologic autophagy inhibition with chloroquine, enhances MRTX1133 efficacy in vitro and in mouse models, including PDXs [174].

### 5.7. New Chemistry-Based Drug Developments

Active-state (RAS(ON)) tri-complex inhibitors (e.g., RMC-6236, RMC-7977) demonstrate pronounced, tumor-selective activity in PDAC by sterically blocking RAS–effector interactions—spanning mutant and wild-type RAS isoforms—offering a route to forestall WT-RAS–mediated rebound [48,108,168,175,176,177]. Pan-KRAS OFF-state inhibitors (BI-2865/BI-2493) broaden coverage across GDP-bound KRAS variants, complementing allele-selective agents and expanding options for heterogeneous PDAC genotypes [178].

Targeted protein degradation is entering clinical trials for KRAS: ASP3082 (a KRASG12D degrader) shows acceptable safety and early activity, while the pan-KRAS PROTAC ACBI3 degrades multiple KRAS mutants, illustrating catalytic elimination as a modality to overcome scaffolding and residual signaling [64,179,180]. Novel agents continue to diversify available options: AZD0022 (G12D-selective, oral) and RMC-5127 (G12V-selective, RAS(ON) tri-complex) extend allele coverage; pan-RAS small molecules (ADT-1004/ADT-007) inhibit nucleotide binding across mutant and WT RAS, priming immune responses in PDAC models; and emergent pan-RAS molecular glues with oral profiles (e.g., RCZY-690/RCZY-680, HZ-V068) report robust CDX activity [77,181,182,183,184,185,186]. Antisense and sequence-selective approaches (e.g., peptide nucleic acid oligomers) provide mutation-directed transcriptional suppression, potentially avoiding cross-resistance from secondary conformational changes and complementing inhibitor-based strategies [187]. One study demonstrated the role of novel CRISPR-CasRx system to control disease progression in PDAC. The CasRx effectively blocked KRAS G12D-signaling pathway in the cancer cells. The silencing of mutant mRNA at the mRNA level was achieved by the CasRx gRNA system, which in turn exhibited a potent anti-tumor effect. Further clinical trials with CasRx is warranted to determine its safety and efficacy [57].

### 5.8. Better Drug Delivery Systems

Nanoparticle platforms-lipid/polymer hybrids, RGD-HSA conjugates, and ligand-targeted constructs-enhance uptake and payload release; co-delivery of siKRAS and erlotinib via chitosan NPs produces synergistic gene silencing and reduced invasion in vitro, while albumin- and RGD-modified carriers improve tumor penetration in PDAC models [188,189,190,191,192].

### 5.9. Use of Advanced Preclinical Models: Organoids, Patient-Derived Xenografts, Single-Cell Genomics to Guide Trials

KRAS-targeted responses differ across model systems: 2D lines under-represent mechanotransduction and TME cues, whereas 3D organoids and xenografts reveal pronounced cytostasis to MRTX1133 and highlight focal adhesion and TME remodeling as modulators of efficacy [160]. Organoids enable high-throughput discovery of drug–gene interactions; isogenic platforms and human PDOs have identified selective vulnerabilities (e.g., KRASG12D dependence on SREBP2-mediated cholesterol biosynthesis), bridging screening hits to translational validation [193].

PDXs and PDX-derived organoids capture MAPK pathway activity independent of KRAS status and model RTK/MAPK-targeted combinations, including pan-RAF + MEK inhibition in KRAS wild-type contexts-a relevant paradigm for KRASWT subsets in trials [128,194,195,196]. Genetically engineered mouse models (KPC, KC) authentically recapitulate PDAC progression, resistance, and TME, and have been indispensable for testing allele-selective inhibitors, RAS(ON) blockade, and IO combinations prior to clinical translation [104,161,197].

Quantitative imaging biomarkers in preclinical PDAC (diffusion-weighted and DCE MRI) detect early tumor cell death and acquired resistance to KRAS within days, offering clinically ready tools to monitor pharmacodynamic effects and adapt therapy in clinical trials [198]. Single-cell and spatial analyses delineate PDAC cell states and immune–stromal crosstalk, clarifying heterogeneity (e.g., classical vs. basal-like subtypes) and identifying adaptive programs and lineage vulnerabilities that should guide stratification and combination design [199].

## 6. Conclusions

Previously, KRAS mutations were considered undruggable. However, trials of KRAS inhibitors, immunotherapies, and vaccines have been conducted in recent years, marking significant progress in this field. However, the only phase 3 clinical trial that gained clinical significance is RASolute 302, which is designed to block RAS signaling in PDAC and is being evaluated for expedited FDA approval in metastatic pancreatic cancer. Current clinical trials have focused on KRAS G12C in PDAC and are in phase 2/3. Studies of G12D- and G12V-targeted therapies remain in phase 1 trials. Advances in ERK inhibition, PI3 inhibition, allosteric modulators, and switch region targeting have been made but remain speculative. The bigger challenge is cross-inhibition or crosstalk, which can lead to signaling through alternative pathways, e.g., MEK inhibition can activate AKT. Many computational approaches have been used to identify new targets, but there has been a limitation in practically identifying potential tight-binding sites for these molecules. Progress has been made in developing KRAS vaccines, biologics such as antibodies and engineered protein inhibitors, and combination therapies combining chemotherapy, KRAS vaccines, and monoclonal antibodies; however, these remain experimental. It is necessary to include more PDAC patients in clinical trials of the above treatments to yield more data on safety, efficacy, and adverse effects in this population.

## Figures and Tables

**Table 1:** KRAS Targeted Agents with Relevance to Pancreatic Ductal Adenocarcinoma (PDAC).

Agent	Mutation Target	Mechanism of Action	PDAC Inclusion (Cohort Type)	Clinical Phase	Key PDAC-Relevant Endpoints
Sotorasib (AMG-510)	KRAS G12C	Covalent KRAS G12C inhibitor (GDP-state)	Yes (basket solid tumor cohorts; limited PDAC patients)	Phase I/II	ORR (%)/DCR (%) = 21%/84% mPFS = 4 mo mOS = 6.9 mo (frontiersin.org)
Adagrasib (MRTX849)	KRAS G12C	Covalent KRAS G12C inhibitor (GDP-state)	Yes (basket cohorts including PDAC)	Phase I–III	ORR (%)/DCR (%) = 33%/100% mPFS = 5.4 mo mOS = 8.0 mo (frontiersin.org)
RMC-6291	KRAS G12C (RAS-ON)	Active-state KRAS G12C inhibitor	Yes (solid tumor escalation and expansion cohorts including PDAC)	Phase I	Safety, PK/PD, preliminary antitumor activity; PDAC-specific efficacy pending.
ARV-806	KRAS G12D	PROTAC-based KRAS G12D degrader	Yes (advanced solid tumors including PDAC)	Phase II	Safety, KRAS degradation biomarkers; clinical PDAC endpoints not yet reported
RMC-6236 (Daraxonrasib)	Multi-KRAS (RAS-ON)	Pan-KRAS active-state inhibitor	Yes (dedicated metastatic PDAC cohorts)	Phase I–III programs	ORR (%)/DCR (%) = 20%/87% mPFS and mOS data maturing. (ir.revmed.com)
RASolute-302	Multi-RAS (RAS-ON)	Active-state multi-RAS inhibitor	Yes (advanced solid tumors including PDAC)	Phase I	Safety, PK, pathway inhibition; PDAC efficacy exploratory
ERAS-007 (HERKULES-3) ± combinations	MAPK pathway alterations	ERK1/2 inhibitor	Yes (CRC and PDAC combination cohorts)	Phase Ib/II	MAPK pathway suppression, ORR with combination therapy; PDAC cohort exploratory
VS-6766 + Defactinib	RAS/MAPK pathway mutations	RAF–MEK clamp + FAK inhibition	Yes (solid tumors including PDAC)	Phase I/II	ORR, PFS, pathway inhibition; limited PDAC-specific data

Abbreviations: PDAC, pancreatic ductal adenocarcinoma; ORR, objective response rate; DCR, disease control rate; PFS, progression-free survival; PDAC response data are from clinical trials and publications as of 2025. Sotorasib and adagrasib data from Phase I/II CodeBreaK-100 and KRYSTAL-1 studies. RMC-6236 early efficacy data reported at ESMO/clinical updates. The remaining data were derived from ClinicalTrials.gov and published clinical trial reports.

## Data Availability

No new data were created or analyzed in this study. Data were obtained from publicly available sources, including peer-reviewed articles identified through Google scholar and trial data available at clinicaltrials.gov.
